# Improving Memory Inhibition: A Study of Retrieval Induced Forgetting, Executive Control, and Chronic Aerobic Exercise

**DOI:** 10.3389/fnbeh.2018.00318

**Published:** 2018-12-19

**Authors:** Concepción Padilla, Pilar Andres, Teresa Bajo

**Affiliations:** ^1^Neuropsychology and Cognition Research Group, Department of Psychology, Research Institute on Health Sciences, University of the Balearic Islands, Palma de Mallorca, Spain; ^2^Instituto de Investigación Sanitaria Islas Baleares, Palma de Mallorca, Spain; ^3^Brain, Mind and Behavior Research Center, University of Granada, Granada, Spain

**Keywords:** aerobic exercise, executive functions, memory, inhibition, RIF, forgetting

## Abstract

Chronic aerobic exercise is being established as a way to enhance executive functions and prevent cognitive decline. In the current study, we are aiming to investigate whether chronic aerobic exercise would also modulate long-term memory retrieval under the context of the Retrieval Practice Paradigm. According to Anderson et al. (1994), the retrieval of relevant information may decrease the access to other related information inducing the failure to remember or forgetting Interestingly, it has been shown (Román et al., 2009) that this process is mediated by the level of attentional resources. In order to test if chronic aerobic exercise benefits attentional resources, we have applied the Dual Retrieval Practice Task. The purpose of this task is to evaluate the Retrieval Induced Forgetting (RIF) effect, which is supposed to index adaptive forgetting. More specifically, the Dual Retrieval Practice Task assesses the effects of memory retrieval on forgetting information directly related to the information that has been previously retrieved, but also studies the involvement of attentional resources on this type of forgetting (retrieval induced forgetting). This task alternates memory retrieval practice with an updating task in order to load attentional resources. Two groups of physically active and sedentary young participants were evaluated. The results showed that while active participants were able to show RIF despite the overload of the attentional resources, sedentary participants were not. These results are discussed in terms of the modulatory role of chronic aerobic exercise on executive control and retrieval induced forgetting.

## Introduction

Long-term and regular aerobic exercise –chronic aerobic exercise– has been established as a factor leading to improvements in executive control (Colcombe and Kramer, 2003; Biddle and Asare, 2011; Bherer et al., 2013; Guiney and Machado, 2013; Cox et al., 2015). Chronic aerobic exercise has also been associated with greater prefrontal (Erickson et al., 2014) and hippocampal (Leavitt et al., 2014) volumes, structures that are involved in executive functions and memory, respectively.

Padilla et al. (2017) investigated to what extent episodic memory would also benefit from chronic aerobic exercise through the improvement of executive functions. They applied a selective attention task where participants were told to remember only those items that were selectively attended. The results showed that those participants who had practiced regular aerobic exercise applied better suppression mechanisms, leading to a lower recognition rate of non-attended items. In a previous study, Padilla et al. (2013) found that active participants were able to inhibit non-relevant motor responses faster than sedentary participants using a strategic stop signal task –a highly demanding dual task-. Finally, Padilla et al. (2014) showed that active participants had a greater working memory span than sedentary participants in a dual working memory task (Unsworth et al., 2005). Taken together, these results may indicate that chronic aerobic exercise has a specific effect on the way active participants deal with highly demanding dual tasks, which requires either suppressing non-relevant information/responses or storing information while they are performing a second task. In order to explore whether the actual effect of chronic aerobic exercise in inhibitory control is related to attentional resources, it was decided to apply the “Dual-Retrieval Practice Task” (D-RP; Román et al., 2009) under the context of the Retrieval Practice paradigm (RP, Anderson et al., 1994). The D-RP task explores the role of attentional resources on exerting suppression of interference –inhibition– in memory, since it combines a working memory task with episodic memory retrieval of competing information.

Within a larger context, inhibition control has been presented as one of the core executive functions (Miyake et al., 2000). Although defining inhibition has not been an elementary task (see Harnishfeger, 1995; Nigg, 2000), some researchers have claimed the existence of a family of different types of inhibitory processes (e.g., Harnishfeger, 1995; Hasher et al., 1999; Nigg, 2000; Friedman and Miyake, 2004). One of the features that has been argued to be crucial in distinguishing between different types of inhibitory tasks is the required level of executive control involved. According to several authors (Harnishfeger, 1995; Nigg, 2000; Friedman and Miyake, 2004; Andrés et al., 2008), inhibitory tasks can be classified into a continuum from very controlled to very automatic, and this also applies to RP. It must be taken into account that these authors (Harnishfeger, 1995; Nigg, 2000; Friedman and Miyake, 2004; Andrés et al., 2008) assume that the type of inhibition that requires a high executive control indirectly demands a high level of attentional resources. Those resources will allow non-relevant information or processes to be filtered out, keep mental representations active while mental operations are being exerted, but also to allocate attention to different tasks if necessary. Thus, controlled inhibition requires a process of collaboration along with working memory (Hasher and Zacks, 1988; Bjork, 1989; Redick et al., 2011).

Returning to the RP paradigm, it is meant to explore forgetting when interference is present. When only certain items from defined list need to be recalled, forgetting the irrelevant items can be a necessary and adaptive process. Under these circumstances, it has been shown that intentionally recalling information may lead to forgetting other information (Wimber et al., 2015). In other words, the very act of remembering information may be one of the major reasons why we forget other information.

The Ordinary Retrieval Practice task (O-RP; Anderson et al., 1994) consists of three phases. First, in the study phase, several lists of category-exemplars (FRUIT-Orange; REPTILE-Snake) are studied. Each category contains multiple exemplars. Second, in the retrieval practice phase, only 50% of the exemplars from one half of the categories must be retrieved with the aid of a cue (FRUIT-Or____). Each exemplar is retrieved a fixed number of times during this phase. Finally, in the recognition phase, all exemplars are presented one-by-one and participants must decide if the exemplar was studied or not in the study phase (see Hicks and Starns, 2004; Gómez-Ariza et al., 2005; Bajo et al., 2006; for recognition based RIF).

The exemplars that were practiced during the retrieval practice phase are considered Rp+. The exemplars that were not retrieved but belonged to the categories practiced in the retrieval phase are called Rp−. Finally, the exemplars that were not retrieved and did not belong to the categories practiced in the retrieval phase are named Nrp. The typical key finding is that the non-practiced (Rp−) items are later less recognized than the control items (Nrp). One of the most plausible explanations is that this retrieval induced forgetting (RIF) effect is the consequence of an inhibitory mechanism that functions at the retrieval phase to reduce the activation of competitive non-target items (Rp−) that interfere with the retrieval of target items (Rp+, Anderson et al., 1994). As a consequence, later in the recognition phase, Rp− items are more difficult to access than Nrp items even though both were equally non-practiced (see Murayama et al., 2014 for a review).

Further studies have explored the extent to which the RIF effect may be modulated by executive resources (Román et al., 2009; Aslan and Bäuml, 2011; Ortega et al., 2012). In this way, Román et al. (2009) designed the “Dual-Retrieval Practice Task” (D-RP) combining a working memory task (remembering five digits or updating a sequence of three numbers) with the retrieval of the previously studied exemplars. They showed that in the dual condition –digit retrieval/updating plus exemplar retrieval–, the RIF effect was absent given that accuracy and response times were similar for Rp− and Nrp items in the recognition phase. This result suggested that some attentional resources are involved in the inhibition of memories at the retrieval phase, despite the lack of intentionality. Subsequently, Ortega et al. (2012) applied the D-RP task in a sample of young and older adults to investigate the effects of aging on the RIF effect. The results showed that there was a RIF effect in the young group when the updating task consisted of three digits, but not when consisting of five digits, confirming the idea that memory inhibition is dependent on executive control. Moreover, RIF was not observed in the older adult group even for the three digits updating D-RP task, revealing that the reduction in executive resources typically observed in aging led to the disappearance of the RIF effect in the D-RP task. Finally, in line with the idea that RIF involved some executive control, Aslan and Bäuml (2011) showed a positive correlation between RIF and working memory capacity in younger adults.

Until now, several studies looking at individual differences in RIF have concentrated on populations that were supposed to show a reduced forgetting effect observed in the RP task (Amir et al., 2009; Soriano et al., 2009; Law et al., 2012; Saunders, 2012; Storm and Jobe, 2012). However, we wanted to investigate whether the RIF effect may be potentiated, especially by a factor (aerobic exercise) that has recently received increased attention in psychological research.

If the type of inhibition involved in the D-RP task can be modulated by executive resources, as has been shown in previous studies (Román et al., 2009; Ortega et al., 2012), then the D-RP task should be sensitive to physical activity, a factor that has been shown to improve executive functions. The hypothesis of the current study is that active participants will display additional executive resources compared to sedentary participants, and this will allow them to deal with the interference induced by the dual-working memory task whereas they suppress the Rp− items. In other words, we predict that physically active participants will display a RIF effect despite being in a dual-task situation, whereas sedentary participants will not.

## Materials and Methods

### Participants

The participants were from the city of Granada,Spain; none of them had a history of mental disorders or physical illnesses incompatible with the study. Written consent was given at the beginning of the experimental procedure, allowing the anonymous data to be used in the study and further publications. All participants were remunerated for their participation in the study. Ethical approval was obtained from the University of Granada Ethics Committee and the study was performed in accordance with the 1964 Declaration of Helsinki and its later amendments.

The participants were 42 young adults, who were divided in two groups according to their fitness level and their frequency of exercise. There were 21 participants in each group (see Table 1 for demographic details). To be included in the active group, participants should have practiced high intensity aerobic exercise (i.e., running, cycling, swimming) for at least the last 7 years, with a frequency of at least 4 h distributed over at least 3 days in a week. This criterion was selected following on previous studies carried out in long-term cardiovascular exercise (e.g., Padilla et al., 2013, 2014; Pérez et al., 2014). The second group, the sedentary participants, must not have exercised for more than 3.5 h per week in the last 4 years and restricted to low-intensity exercise (i.e., walking). The sedentary group must also not have practiced aerobic exercise with a frequency higher than 6 h per week during their childhood and early adolescence (0–14 years), taking into account that physical education is taught 3 days a week within the Spanish education system.

**Table 1:** Demographic data of the active and sedentary participants.

		Active	Sedentary	Group differences
		Count/Mean	Count/Mean	*p* values
Participants		21	21	
Gender	Male	11	7	0.21^b^
	Female	10	14	
Age		23.90 (2.61)	24.86 (3.41)	0.32^c^
Cardiovascular Level (20-shuttle run test)		31.29 (11.33)	16.73 (7.63)	0.00^∗∗c^
Total hours of exercise		5775.97 (2393.47)	696.62 (850.78)	0.00^∗∗c^
Years of education		17.14 (2.92)	17.00 (4.16)	0.89^c^
Education level	Compulsory education	0	2	0.29^b^
	College	5	7	
	Graduate	12	7	
	Postgraduate	4	5	
Vocabulary (WAIS)		47.76 (4.97)	48.55 (5.29)	0.63^c^
Raven Accuracy		9.38 (3.32)	9.67 (4.08)	0.81^c^
Total CRIq		88.86 (15.91)	94.29 (9.66)	0.19^c^
	CRIq education	99.90 (10.25)	98.33 (12.38)	0.66^c^
	CRIq profession	93.24 (3.92)	93.33 (4.66)	0.92^a^
	CRIq leisure	97.69 (7.49)	96.40 (6.98)	0.60^c^
Spoken languages	Only L1	6	6	0.89^b^
	L1 and L2 (co-official languages)	8	9	
	L1, L2, and L3 (including one co-official language)	4	6	
	More than 2 languages	0	0	
Depression level	Normal mood fluctuations	20	18	0.29^b^
	Mild mood disturbance	1	3	
	Intermittent depression states	0	0	
STAI trait	PD ≤ 25	15	9	0.10^b^
	PD 26–50	5	5	
	PD 51–75	1	5	
	PD > 76	0	2	

*^a^Mann–Whitney test for non-normal distributions. ^b^Pearson’s chi-square test for categorical variables. ^c^T-tests. ^∗∗^ p <0.001.*

In order to make sure that both groups had different cardiovascular levels, the 20 m shuttle run test (Léger et al., 1988) was applied.

Moreover, as pre-existing differences between participants belonging to each group might occur, an extensive evaluation of the following aspects was carried out: (a) Years and level of education, profession, and leisure activities using the Cognitive Reserve Index questionnaire (CRIq, Nucci et al., 2012); (b) Crystallized (Wechsler Adult Intelligence Scale, WAIS-III) and fluid (Raven’s Advanced Progressive Matrices (Raven et al., 1996) intelligence; (c) Languages spoken; (d) Depression (Beck Depression Inventory; Beck et al., 1996); and (e) Anxiety (the State-Trait Anxiety Inventory; Spielberger et al., 1970).

### Procedure

Participants were recruited through different advertisements at the University of Granada and were asked to complete two online questionnaires to quantify their aerobic exercise frequency and cardiovascular level, in addition to education and leisure activities through the CRIq. A question about fluent spoken languages was included in the CRIq, since it has been shown that bilingualism enhances cognitive reserve and executive functions (Ljunberg et al., 2013; Bialystok et al., 2014).

Participants were interviewed by telephone by an experienced neuropsychologist to complete the information required. Subsequently, participants were informed about the experiment and asked to complete several online questionnaires to assess anxiety levels (the State-Trait Anxiety Inventory; Spielberger et al., 1970) and depression (Beck Depression Inventory; Beck et al., 1996) since these mental disorders may affect performance in executive functions tasks (Pacheco-Unguetti et al., 2010; Snyder, 2013). Participants subsequently attended the Brain, Mind and Behavior Research Center at the University of Granada to perform the cognitive tasks and the cardiorespiratory capacity test.

### Cognitive Tasks

Participants carried out three cognitive tests in a quiet room during a 2 h session. The first 30 min were dedicated to introducing and describing the tasks and completing informed consent. Participants then began the cognitive-task session, where the Dual Retrieval Practice Task (D-RP; Román et al., 2009; Ortega et al., 2012) was performed in addition to the two intelligence tests. All tests with the exception of the vocabulary scale were administered using a computer. Raven’s Advanced Progressive Matrices (Raven et al., 1996) and D-RP (Ortega et al., 2012) were designed and displayed using E-Prime 2.0 software (Psychology Software Tools, Pittsburgh, PA, Schneider et al., 2002).

To begin, participants performed the D-RP task, followed by the Wechsler Adult Intelligence Scale Vocabulary subtest (Wechsler, 1999). Next, a computerized version of Raven’s Advanced Progressive Matrices (Raven et al., 1996) was applied to obtain a measure of fluid intelligence. Only 18 items from this test were presented, so the time of the session could be shortened, with the time limit set to 20 min.

#### Dual Retrieval Practice Task (D-RP)

**FIGURE 1:**
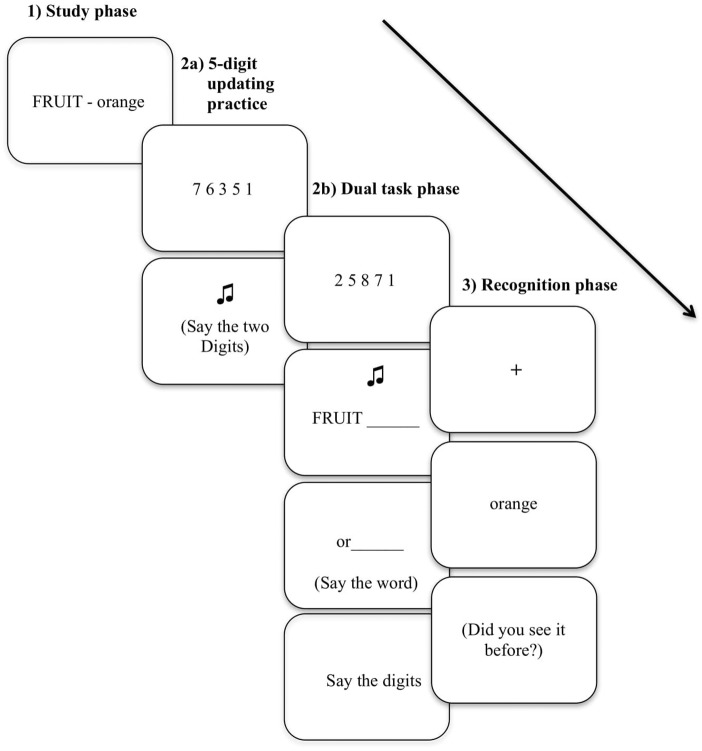
Dual retrieval practice task. (1) Study phase: category–exemplar pairs were shown to be studied. (2a) Five-digit updating practice task: a digit sequence appeared on the screen, followed by a tone of high or low frequency indicating the numbers that had to be said aloud. (2b) Retrieval phase: the updating and the retrieval of Rp+ tasks were combined. (3) Recognition phase: the exemplars from the study phase were presented interleaved with new exemplars. See text for detailed information.

The D-RP task was the same as applied in the studies by Román et al. (2009) and Ortega et al. (2012) where a 5-digit updating task was concurrent with RP. This task was used as an alternative to the O-RP task because previous research has shown that it is more sensitive to individual differences in executive control (Ortega et al., 2012). There were four counterbalanced versions of the same task and participants were randomly assigned to one of them. The experiment had three phases: (1) category-exemplar study, (2a) 5-digit-updating practice (2b) dual task: 5-digit updating and retrieval practice; and (3) recognition (see Figure 1).

##### Materials

The materials were identical to those used by Román et al. (2009) and Ortega et al. (2012). Forty-eight category-exemplars pairs were drawn from Battig and Montague (1969) that in turn were divided into eight semantic categories. Two of these categories were fillers intending to control primacy and recency effects. Each category was composed of six exemplars. One constraint was that none of these six exemplars within the category started with the same first two letters. Six blocks of six exemplars each were created; each exemplar was from a different category.

##### Study phase

Participants first read the instructions and then six blocks of 8 category-exemplar pairs were presented: two filler categories plus six experimental ones. The order of these blocks and the exemplars presented into each block were randomized among the participants. Each category-exemplar pair was shown for 5 s at the centre of the screen for encoding. Primacy and recency effects were controlled adding category-exemplar pairs from the two filler categories at the beginning and the end of the study phase. The study phase lasted 4 min plus the time taken to read and understand the instructions.

##### Dual task: five-digit-updating and retrieval-practice phase

Just after the study phase, a 5-digit updating task trial was performed, so the participants could practice this task. Fourteen sequences of five digits were presented for 5 s each at the centre of the screen. Every 5-digits sequence was followed by another screen with a 100 ms high or low frequency tone. The participants had to keep the 5-digits sequence in mind and after hearing the tone, say aloud the two smallest numbers if the tone was high, or the two biggest digits if the tone was low. The participants had 5 s to respond. After this trial, the participants were presented with the dual task, where the 5-digit updating and the retrieval practice tasks (see Figure 1) were combined. There were four practice trials to facilitate the participants’ understanding of the task before starting. In the part of the retrieval practice task, half of the six studied categories were presented. Of those categories, just half of the exemplars from each category were shown. These practiced items are considered Rp+, while exemplars from the practiced categories that did not appear in this phase were considered Rp−. In this dual task, the participants were first presented with a 5-digit sequence for 5 s with the instructions to remember the numbers since they would have to be recalled later in the test. Each sequence was followed by a tone and a screen presenting the category cue in capital letters that remained for 2 s. Following on, the two first letters of the exemplar were shown on a subsequent screen until the participant responded, with a limit of 2 s. The two letters were written in lower case and were followed by a black line (i.e., or_______). Participants had to complete the exemplar immediately by saying it aloud. Next, another screen was presented for 5 s with an instruction requiring the participants to say the two smallest or biggest digits from the sequence, according to the tone the participants had heard before. After 5 s, a white screen appeared for 2 s, so participants had in total a maximum of 7 s to say aloud the numbers. Responses to exemplars and digits were written and recorded by the researcher. Every category-exemplar pair appeared randomly three times in the retrieval phase interleaved with other pairs. A category-exemplar pair from a filler category separated every repetition block; also filler items were included at the beginning and at the end of this phase. In total, three blocks of nine Rp+ items plus six fillers each were presented. The total duration of this phase was approximately 20 min.

##### Recognition phase

In this phase, all the exemplars studied at the beginning of the study were presented along with new exemplars. Participants were requested to say aloud as rapidly and accurately whether the exemplar shown was new or old. As mentioned above, among the studied items were Rp+, Rp−, but also the items from which neither the exemplar nor their category had been practiced in the retrieval practice phase (Nrp). Between the new items, there were items belonging to: (a) the practiced categories, (b) unpracticed categories, and (c) new categories (see Figure 1). In this recognition phase there were two blocks: Rp− and Nrp were presented first interleaved randomly with new items; and later in a second block, Rp+ interleaved with new items were shown. This order was aimed at preventing blocking of the critical Rp− items from Rp+ items (Ortega et al., 2012). The task started with a fixation cross that lasted 500 ms, later an exemplar was presented in lower case for 3 s and finally a white screen appeared for 1 s. The participants had to respond with “new” or “old” as soon as the exemplar appeared and had a maximum of 4 s. The total duration of this phase was approximately 4 min.

### Cardiorespiratory Capacity Test

Participants performed the 20 m shuttle run test in order to test their basal VO_2_ max. This cardiovascular test was intentionally scheduled to follow the cognitive tasks to avoid the acute effects of aerobic exercise on cognition, as the objective of the study is to measure the effect of long-term aerobic exercise on the brain under a developmental perspective. The 20 m shuttle run test (Léger et al., 1988) was applied to measure the participant’s cardiovascular level. This test estimates the maximal oxygen uptake (VO_2_ max) from the speed that the participants have reached during a physically demanding test. Participants have to do shuttle runs on a 20 m course crossing a line before a recorded signal sounds. The frequency of the signal increases as the test progresses, requiring the participants to increase speed. As the participants run, they reach different speed stages. The number of the last stage achieved is used to estimate VO_2_ max. The test showed a high validity when it was compared with the maximal multistage treadmill test as well as a high test–retest reliability (Léger et al., 1988).

### Statistical Analyses

#### Demographic Data

Pearson’s chi-square tests were used to check for significant differences in categorical variables, such as gender, education level, spoken languages, evidence of depression, and levels of anxiety. The Mann–Whitney *U*-test was used with the variable “CRIq profession,” as it did not follow the normal distribution. Independent samples *t*-tests were used for those continuous demographic variables following the normal distribution.

#### Dual Retrieval Practice

Initially, digit accuracy was calculated as the proportion of digits correctly recalled.

Secondly, word accuracy was calculated taking into account only the Rp+ exemplars correctly recalled. Finally, *t*-tests were applied to compare digit and word accuracy between groups.

#### Recognition Phase

Accuracy in the recognition phase was calculated using corrected hits. Corrected hits are obtained by subtracting false alarms from hits in the corresponding conditions. Thus, first, false alarms caused by new items that belonged to practiced categories (Rp false alarms) were subtracted from hits in Rp−; second, false alarms caused by new items that belonged to practiced categories (Rp false alarms) were subtracted from hits in Rp+; and finally, false alarms caused by new items that belonged to studied, but not practiced categories (Nrp false alarms) were subtracted from hits in Nrp.

The RIF effect was measured as the difference between the Rp− and Nrp conditions. A 2 (group: active vs. sedentary) × 2 (condition: Rp− vs. Nrp) repeated measures ANOVA was calculated on the corrected hits. Pairwise comparisons were carried out – which apply the Bonferroni correction for multiple comparisons- to explore the condition × group interaction.

The facilitation effect was measured as the difference between Rp+ and Nrp items. A 2 (group: active vs. sedentary) × 2 (condition: Rp+ vs. Nrp) repeated measures ANOVA was calculated on the corrected hits. As previously, pairwise comparisons applying the Bonferroni correction for multiple comparisons were carried out.

## Results

### Demographic Data

Table 1 shows the mean and standard deviations for each of the demographic variables. There was a similar number of males and females in each group [χ^2^(1) = 1.56, *p* = 0.21]. Active and sedentary groups did not differ in age [*t*(40) = 1.02, *p* = 0.32, *d* = 0.32], or crystallized (WAIS) [*t*(40) = 0.49, *p* = 0.63, *d* = 0.15] or fluid (Raven) [*t*(40) = 0.25, *p* = 0.81, *d* = 0.08] intelligence.

Moreover, active and sedentary groups did not differ either in number of years of education [*t*(40) = 0.13, *p* = 0.89, *d* = 0.04], education level [χ^2^(3) = 3.76, *p* = 0.29], general Cognitive Reserve Index questionnaire (CRIq, Nucci et al., 2012) [*t*(40) = 1.34, *p* = 0.19, *d* = 0.42] or specific CRIq indexes: (a) education [*t*(40) = 0.45, *p* = 0.66, *d* = 0.14], (b) profession [*U* = 216.50, *z* = -0.10, *p* = 0.92] and (c) leisure activities [*t*(40) = 0.53, *p* = 0.60, *d* = 0.17]; number of languages spoken [χ^2^(2) = 0.23, *p* = 0.89], depression [χ^2^(1) = 1.11, *p* = 0.29] or anxiety levels [χ^2^(3) = 6.17, *p* = 0.10].

Importantly, active and sedentary participants differed significantly in terms of cardiovascular level [*t*(40) = 4.80, *p* < 0.001, *d* = 1.52] and exercise frequency [*t*(40) = 9.87, *p* < 0.001, *d* = 3.12].

### Dual Retrieval Practice

Accuracy (mean and standard deviation) for sedentary and active participants during the dual retrieval practice is presented in Table 2. *T*-tests comparing the proportion of digits accuracy indicated that active and sedentary participants recalled a similar proportion of digits [*t*(40) = 0.35, *p* = 0.73, *d* = 0.11]. The proportion of word accuracy was also similar between groups [*t*(40) = 1.76, *p* = 0.09, *d* = 0.56].

**Table 2:** Mean proportion of correctly retrieved words and mean proportion of correctly recalled digits in the dual phase.

	Active	Passive
Word accuracy	0.66 (0.17)	0.74 (0.14)
Digit accuracy	0.89 (0.10)	0.90 (0.09)

### Recognition

Corrected hits, hits, and false alarms (mean and standard deviation) are presented in Table 3. The results from the repeated measures ANOVA indicated that active and sedentary participants did not differ in terms of false alarms [*F*(1, 40) = 0.70, *MSE* = 0.22, *p* = 0.41, η^2^*_p_* = 0.02]. The number of Rp and Nrp false alarms was not significantly different [*F*(1, 40) = 3.21, *MSE* = 0.07, *p* = 0.08, η^2^*_p_* = 0.07], and no interaction between the conditions were found [*F*(1, 40) = 3.16, *MSE* = 0.07, *p* = 0.08, η^2^*_p_* = 0.07].

**Table 3:** Mean proportions of corrected hits, hits, and false percentage (standard deviation in brackets) per group and type of item.

			Type of item		
Group	Variable	Participants	Rp+	Rp−	Nrp
Active	Corrected hits	21	0.65 (0.29)	0.36 (0.30)	0.58 (0.23)
	Hits		0.84 (0.19)	0.57 (0.27)	0.66 (0.20)
	False alarms		0.19 (0.20)		0.08 (0.13)
Sedentary	Corrected hits	21	0.81 (0.21)	0.54 (0.29)	0.54 (0.24)
	Hits		0.91 (0.12)	0.64 (0.19)	0.64 (0.20)
	False alarms		0.10 (0.16)		0.10 (0.15)

*Corrected hits are obtained subtracting false alarms from hits in the corresponding conditions.*

**FIGURE 2:**
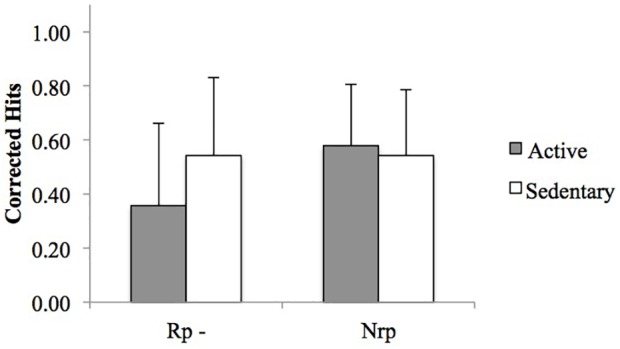
Mean Rp− and Nrp corrected hits per group. Error bars indicate the standard deviation. Rp−: exemplars not retrieved during retrieval practice but belonging to the categories practiced in the retrieval phase. Nrp: exemplars from different categories from the exemplars that were practiced.

The repeated measures ANOVA calculated for the Rp – and Nrp corrected hits (see Figure 2) revealed a significant effect of condition [*F*(1, 40) = 5.78, *MSE* = 0.26, *p* = 0.02, η^2^*_p_* = 0.13], showing that Nrp items (*M* = 0.56, *SD* = 0.23) were better retrieved than Rp− items (*M* = 0.45, *SD* = 0.31). There was no significant group effect [*F*(1, 40) = 1.19, *MSE* = 0.12, *p* = 0.28, η^2^*_p_* = 0.03], but a significant condition × group interaction [*F*(1, 40) = 5.88, *MSE* = 0.26, *p* = 0.02, η^2^*_p_* = 0.13] was observed.

The results from the pairwise comparisons revealed that active participants recognized significantly fewer Rp− (*M* = 0.36, *SD* = 0.3) items than the sedentary (*M* = 0.54, *SD* = 0.29) (*p* = 0.048). There was, however, no significant difference between active and sedentary participants for the Nrp exemplars (*p* = 0.62). *T*-tests carried out to study forgetting effect indicated that active participants showed significant RIF [*t*(20) = 3.81, *p <* 0.001, *d* = 1.70], while sedentary participants did not [*t*(20) = 0.01, *p* = 0.99, *d* = 0.00].

**FIGURE 3:**
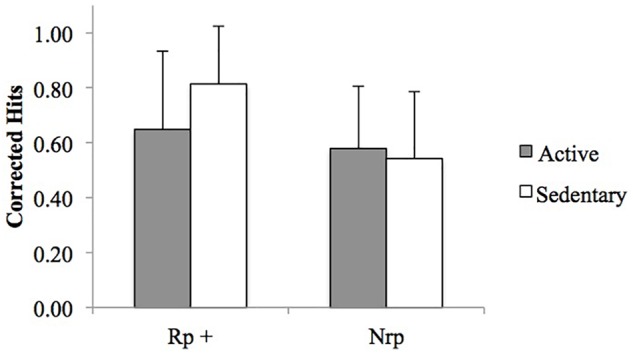
Mean Rp+ and Nrp corrected hits per group. Error bars indicate the standard deviation. Rp+: exemplars practiced during the retrieval practice phase. Nrp: exemplars from different categories from the exemplars that were practiced.

Rp+ and Nrp corrected hits (means and standard deviations) are presented in Table 3 and Figure 3. The repeated measures ANOVA on these variables revealed a significant condition effect [*F*(1, 40) = 15.64, *MSE* = 1.22, *p* < 0.001, η^2^*_p_* = 0.28], showing that Rp+ items (*M* = 0.73, *SD* = 0.26) were better recognized than Nrp items (*M* = 0.56, *SD* = 0.23). The group effect was not significant [*F*(1, 40) = 1.09, *MSE* = 0.43, *p* = 0.30, η^2^*_p_* = 0.03]. The group × condition interaction was, however, significant [*F*(1, 40) = 5.49, *MSE* = 0.43, *p* = 0.02, η^2^*_p_* = 0.12]. Pairwise comparisons revealed that sedentary participants recognized more Rp+ items (*M* = 0.81, *SD* = 0.21) than the active participants (*M* = 0.65, *SD* = 0.29) (*p* = 0.039), whereas they did not differ in Nrp recognition (*p* = 0.62).

## Discussion

The aim of this study was to investigate whether the inhibitory processes involved in the retrieval of competitive elements from memory might be positively modulated by chronic aerobic exercise through attentional resources. The main prediction was that physically active participants would show RIF under a high demanding executive task –D-Rp−, whereas sedentary participants would not.

The results first indicated that active and sedentary participants did not differ in factors that could have improved executive control in active participants such as bilingualism, education, crystallized and fluid intelligence or age. This equality between sedentary and active participants on non-manipulated and potentially confounding variables makes potential group differences in D-RP statistically valid.

There remains, however, some putative uncontrolled variables that might affect RIF. For example, some studies have described self-control and self-regulation differences between active and sedentary participants (Audiffren and André, 2015; Diamond and Ling, 2016, 2018) and this might mediate the observed RIF differences between groups. This would be an empirical question that could be investigated in future studies.

As usually observed in the D-RP task (Ortega et al., 2012), participants did not differ in performance during the retrieval-practice phase. However, active and sedentary groups showed different patterns of recognition. First, while active participants showed a significant RIF effect, the sedentary group did not. Active participants recognized significantly fewer Rp− items than Nrp. Also, active participants recognized fewer Rp− than sedentary participants. Since previous studies have revealed that physically active participants showed improved executive functions (Guiney and Machado, 2013; Padilla et al., 2013, 2014; Pérez et al., 2014), it is likely that active participants were able to suppress Rp− memory traces at retrieval practice despite the concurrent working memory load (updating task). The absence of differences in the digit-recall task –when performed before the dual task– and the similar levels of the Nrp recognition suggest that physical activity specifically modulates the ability to inhibit competing information when executive control is taxed by dual tasking. Therefore, in line with previous studies (Padilla et al., 2013, 2014, 2017; Pérez et al., 2014) our results indicate that physical activity selectively affects inhibitory control through attentional resources.

It is worth noting that the pattern of results revealed by sedentary participants in forgetting was as expected in young adults. Ortega et al. (2012) showed that young participants, when carrying the D-RP task with 5 digits, had an absence of RIF effect. The authors explained this finding by the fact that divided attention caused such a reduction in attentional resources that active suppression of Rp− interference during retrieval could not be undertaken. Similarly, the sedentary participants from the current study did not have enough attentional resources to suppress Rp− interference while dealing with the working memory –updating– task. It can be argued that the chronic practice of physical activity has led to an increase in attentional resources in the active participants, making it possible for them to suppress the interference from the Rp− at the retrieval phase.

Alternatively, the differences in the recognition of the Rp+ items for the sedentary and active group could result from the fact that they were presented in the final part of the recognition phase and after Rp− and Nrp items had been presented. Thus, during the first block of the recognition phase, the retrieval of the Rp− may cause inhibition of the competing Rp+, and this in turn will produce lower recognition of the Rp+ items in the recognition block. Considering sedentary participants demonstrate, in general, less inhibitory capacities, the RP+ items will not be inhibited during the first recognition phase and therefore facilitation is still present for the practiced items. This cost-benefit interplay has been previously illustrated and discussed (Anderson and Levy, 2007).

## Conclusion

The data suggest that greater attentional resources in active participants result in a greater ability to suppress interference when working memory is loaded in the dual-RP updating task. This leads to the understanding that inhibition during retrieval is susceptible to modification by aerobic exercise through the mediation of attentional resources that facilitates divided attention between two tasks. The previous results (Padilla et al., 2013, 2014, 2017; Pérez et al., 2014) were replicated and extended by showing that a long-term routine of physical activity is associated with a improved ability to deal with a demanding dual task such as the D-RP. It would be interesting to corroborate the results with an aerobic exercise intervention, since cross-sectional studies do not allow establishing causal relationships.

In this regard, long-term cardiovascular exercise is accepted as an intervention that benefits cognition since early adulthood. Its effect on attentional resources is reflected in different cognitive functions. These findings may convince policymakers of the importance of exercise and promote the lifelong practice of cardiovascular exercise.

## Data Availability Statement

The raw data supporting the conclusions of this manuscript will be made available by the authors, without undue reservation, to any qualified researcher. Requests to access the datasets should be directed to Dr. Concepcion Padilla at cfp31@medschl.cam.ac.uk.

## Author Contributions

TB, PA, and CP designed the study. CP wrote the paper, analyzed the data, recruited and applied the tests to the participants. PA and TB gave advice and ideas on the analyses and the interpretation of results and helped on the writing of the manuscript.

## Conflict of Interest Statement

The authors declare that the research was conducted in the absence of any commercial or financial relationships that could be construed as a potential conflict of interest.

## Abbreviations

CRIq: Cognitive Reserve Index questionnaire
D-RP: Dual-Retrieval Practice Task
Nrp: The exemplars that were not retrieved and did not belong to the categories practiced in the retrieval phase are named
RIF: Retrieval Induced Forgetting
RP: Retrieval Practice paradigm
Rp+: The exemplars that were practiced during the retrieval practice phase
Rp−: The exemplars that were not retrieved but belonged to the categories practiced in the retrieval phase
